# Manual of the Diseases of Infants and Children

**Published:** 1911-08

**Authors:** 


**Manual of the Diseases of Infants and Children, John Rurăh,** M.D., Baltimore; third edition, 1911, W. B. Saunders Co., Philadelphia. Five hundred and thirty-four pages, 173 illustrations, $2.50.

This is a small book, bound in flexible covers, containing in condensed form a vast amount of information concerning not only the diseases of infants and children but statistics of growth, dietetics, general hygiene, therapeutics, useful hints for securing the necessaries of a complete nursery for the poor and even a list of pediatric medical journals. We would advise especially the careful perusal of the general discussion of the physiology of infancy, of the diseases of the new born and such other chapters as are readily recognised to be out of the beaten track.

Without wishing to appear captious, we would plead for an additional H in "rachitis," for a reduction of the meat ration for young school children below half a pound a day, for a reasonable use of nuts, candy, bananas and simple forms of cake like cookies, for a more cautious use of alcohol in the therapeutics of children, and for the insertion of metric dosage.

While, at first glance, the work impresses one as adapted especially for the undergraduate student and for ready reference by the busy practitioner, it is really very complete and thorough, its brevity being due to careful condensation and the omission of unessential discussions.

